# New-onset disability risk prediction model for chronic respiratory disease patients: the first longitudinal evidence from CHARLS

**DOI:** 10.3389/fmed.2025.1545387

**Published:** 2025-05-20

**Authors:** Xuanna Zhao, Jiahao Cao, Yunan Wang, Jiahua Li, Xianjun Mai, Youping Qiao, Jinyu Liao, Min Chen, Dongming Li, Bin Wu, Dan Huang, Dong Wu

**Affiliations:** Department of Respiratory and Critical Care Medicine, Affiliated Hospital of Guangdong Medical University, Zhanjiang, China

**Keywords:** predictive model, disability, chronic respiratory diseases, nomogram, CHARLS

## Abstract

**Background:**

Although studies have explored the factors influencing the occurrence of disability, predictive models for disability risk in the chronic respiratory diseases (CRD) patient population remain inadequate.

**Methods:**

This study employed baseline data from the 2015 China Health and Retirement Longitudinal Study (CHARLS) to select 803 CRD patients without disabilities, who were then followed for 3 years to observe the emergence of new disabilities. Least Absolute Shrinkage and Selection Operator (LASSO) regression analysis was applied to identify risk factors associated with the onset of disability. Ultimately, multivariable logistic regression analysis pinpointed four critical predictive factors: marital status, self-perceived health, depressive symptoms, and age, which were subsequently incorporated into a nomogram model. The model’s predictive efficacy was evaluated using the receiver operating characteristic curve (ROC), calibration curve, and decision curve analysis (DCA).

**Results:**

During the 3-year follow-up, 196 patients developed new disabilities, yielding an incidence rate of 24.41%. The model evaluation results revealed that area under the curve (AUC) for the training set was 0.724 (95% confidence interval [CI]: 0.676-0.771), and the AUC for the test set was 0.720 (95% CI: 0.641-0.799), demonstrating high accuracy, sensitivity, and specificity. The calibration curve confirmed that the predicted results aligned closely with the actual outcomes, while the DCA analysis illustrated that the model provided substantial net benefits in clinical decision-making, effectively identifying high-risk patients.

**Conclusion:**

The nomogram model developed in this study effectively predicts the risk of new disability occurrence in CRD patients within 3 years. By identifying high-risk patients at an early stage, this model provides scientific evidence for early intervention and health management in CRD patients.

## 1 Introduction

Chronic respiratory diseases (CRD) encompass a range of conditions that impact the airways and various lung structures, constituting some of the primary contributors to global morbidity and mortality. Among these, Chronic Obstructive Pulmonary Disease (COPD) and Asthma are the most prevalent. As individuals with chronic respiratory conditions age, their overall health typically declines, particularly impairing their ability to perform essential daily activities, such as bathing, laundry, and eating (1). This deterioration in functional capacity is not only correlated with a diminished quality of life but also associated with elevated hospitalization rates and an augmented risk of mortality. Based on recent report, CRD was responsible for 103.5 million (94.8–112.3) Disability-Adjusted Life Years (DALYs) constituting 4.1% (3.7–4.4%) of global DALYs for all causes (2). What’s more, the burden of CRD is particularly pronounced during episodes of acute exacerbation (3), which can lead to significant psychological stress that further complicates disease management. Patients with CRD often experience higher levels of psychological distress and exhibit poorer social functioning compared to the general population (4).

Disability is typically defined as the difficulty in carrying out fundamental tasks, such as activities of daily living (ADL) and instrumental activities of daily living (IADL) (5, 6). Limitations in ADL or IADL not only hinder the ability of older adults to live independently but also substantially diminish their quality of life. A Japanese study conducted in Japan demonstrated that individuals with lower ADL scores exhibited a mortality rate twice as high as those with higher scores (7). Previous research has identified numerous risk factors for disability among middle-aged and older adults, including advanced age, female gender, chronic diseases, limited physical activity, lack of social engagement, poor self-perceived health, dyslipidemia, smoking, abnormal body mass index, depressive symptoms, and memory decline—factors that are all strongly associated with the onset of disability (8–12). Nevertheless, research investigating the connection between CRD and the risk of disability remains scarce, with limited exploration of the specific risk factors for disability within the CRD population.

Functional limitations and the resulting disabilities not only cause a profound decline in an individual’s quality of life but also place a significant economic strain on society (13). Consequently, the early screening of high-risk populations for timely intervention to prevent the onset and progression of disability is of paramount importance. Risk prediction models, as invaluable tools, can be employed to assess the disability risk in patients with CRD. Previous research has predominantly concentrated on healthy elderly populations. For instance, Han et al. developed and validated a disability prediction model for healthy elderly individuals in China. Their study identified several factors—such as age, marital status, napping habits, white blood cell count, systolic and diastolic blood pressure, right-hand grip strength, respiratory function, memory, standing balance, and depressive symptoms—as independently linked to the onset of disability (1).

However, the majority of existing prediction models are primarily based on healthy populations, with limited efforts made to construct risk prediction models for identifying individuals at high risk for new disability onset within CRD patient populations. Our study seeks to investigate the key factors associated with the occurrence of disability and integrate them into a nomogram to create a disability prediction model specifically for CRD patients, with the goal of providing a more precise tool for assessing disability risk in this population.

## 2 Materials and methods

### 2.1 Study population

**FIGURE 1:**
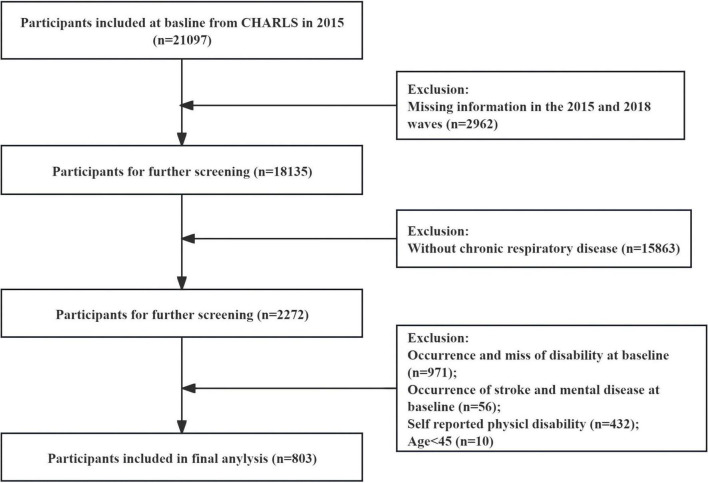
Participant selection process flowchart.

This study was a retrospective cohort study within the general population of China Health and Retirement Longitudinal Study (CHARLS). CHARLS is an ongoing longitudinal survey in China, dedicated to exploring the health, economic circumstances, and retirement status of the elderly population. Data from the 2015 and 2018 waves of CHARLS were collected and analyzed in this study, with access available publicly at http://charls.pku.edu.cn. The CHARLS protocol received approval from the Ethical Review Committee of Peking University (Approval number: IRB00001052-11015), and written informed consent was obtained from each participant (14). We incorporated 18,135 participants from the 2015–2018 study wave, of whom 803 met the criteria for model development and validation. These individuals had reported no disabilities in the 2015 wave. As shown in Figure 1, exclusion criteria were as follows: (1) participants under the age of 45; (2) participants who did not self-report having chronic respiratory diseases in the 2015 survey; (3) participants with missing physical function scores (ADL and IADL scores) in the 2015 survey; and (4) participants who self-reported having physical disabilities, stroke, or mental diseases.

### 2.2 Chronic lung disease

The outcome variable focused on the incidence of CRD, including chronic bronchitis, emphysema, pulmonary heart disease, and asthma, as recorded in the 2015 CHARLS database. (1) Participants were asked the following questions: “Has a doctor ever informed you that you have CRD, such as chronic bronchitis or emphysema, or even pulmonary heart disease (excluding tumors or cancer)?” and “Has a doctor ever informed you that you have asthma?” Participants who responded affirmatively to either question were classified as having CRD.

### 2.3 Assessment of new-onset disability

Disability was evaluated using assessments of ADL and IADL (5, 6). ADL evaluated participants’ capability to carry out essential daily tasks, including dressing, bathing, eating, getting out of bed, using the toilet, and managing bladder and bowel functions. IADL, conversely, evaluated their capacity to perform more intricate tasks, such as household chores, cooking, shopping, financial management, making phone calls, and taking medications. The participants’ responses were classified into four distinct levels: (1) No difficulty; (2) Some difficulty but can manage independently; (3) Difficulty requiring assistance; and (4) Unable to perform the activity. Each ADL/IADL item was scored as 0 if the participant had no difficulty and 1 if any difficulty or inability was reported. The overall score was determined by aggregating the six items, with participants being grouped into two categories based on the score: (1) non-disability (ADL/IADL score = 0) and (2) disability (ADL/IADL score ≥ 1) (15). In the study, participants with disabilities as of 2015 were excluded. If a patient was subsequently assessed as disabled after this year, they were included in the research according to our criteria until the follow-up period ending in 2018.

### 2.4 Sociodemographic and behavioral characteristics

The demographic factors encompass gender, age, marital status, education level, residence, and retirement status. Gender is categorized as male or female. Marital status is considered “married” if the participant is currently married and living with their spouse, and “unmarried” if the participant is separated, divorced, widowed, or has never been married. Education level is classified as “Illiterate,” “Primary school,” “Middle school,” or “High school or beyond.” Residence is classified as either urban or rural. Retirement status is categorized as “Yes” or “No.” Behavioral factors include smoking history, drinking history, and nighttime sleep duration. Both smoking and drinking histories are recorded as “Yes” or “No.” Data regarding total nighttime sleep duration are gathered through the question: “What was your average nightly sleep duration (in hours) over the past month?” Sleep duration is defined as the actual time the participant spends in bed during the night.

### 2.5 Health status

Drawing upon previous research and our expertise (1, 16, 17), the potential factors selected for predicting disability include a history of chronic diseases (hypertension, diabetes, cancer, heart disease, arthritis, dyslipidemia, liver disease, kidney disease, digestive system diseases), self-perceived health status, systolic blood pressure, diastolic blood pressure, grip strength, body mass index (BMI), waist circumference, peak expiratory flow rate, depression, memory, and cognitive function. Chronic diseases are self-reported diagnoses and are defined as “Yes” or “No.” The term “mental diseases” is defined as “whether a doctor has ever informed you of having emotional, nervous, or psychiatric problems.” Self-perceived health status is categorized as “Good,” “Fair,” or “Poor.” Blood pressure is evaluated using the Omron HEM-7200 monitor, and the average of three readings for both systolic and diastolic pressures is recorded. Grip strength is measured twice for each hand using a dynamometer, and the average grip strength of the right hand is used to reflect overall grip strength. BMI is calculated based on height and weight. Waist circumference is gauged using a flexible measuring tape. Respiratory function is measured three times using a peak flow meter, with the maximum peak expiratory flow rate recorded. The Chinese version of Center for Epidemiologic Studies Depression Scale-10 item (CES-D10) from the Epidemiological Research Center website is used to assess depression symptoms. The CES-D10 consists of 10 items, with each item scored from 0 to 3, reflecting a range from “none” to “almost every day.” The total score spans from 0 to 30, with higher scores signifying more severe depressive symptoms. Cognitive function includes visual-spatial skills, memory, orientation, and attention. Visual-spatial skills are assessed by drawing two overlapping pentagons; one point is given for a correct drawing, and zero for an incorrect one. Memory is assessed by the average score of immediate and delayed recall of 10 Chinese words, with one point granted for each word correctly remembered. Attention and orientation are evaluated using the Telephone Interview for Cognitive Status (TIC-10), which derives the score from responses to questions regarding the year, month, day, week, and season, as well as by repeatedly subtracting 7 from 100 (up to five times). One point is granted for each correct response, with the total score ranging from 0 to 10. The aggregate of the aforementioned dimensions forms the overall cognitive function score, ranging from 0 to 21, with higher scores indicating enhanced cognitive function.

### 2.6 Statistical analysis

Continuous variables are presented as mean ± standard deviation (SD) or median (interquartile range [IQR]), and comparisons were made using the Student’s *t*-test or the Wilcoxon rank-sum test. Categorical variables are expressed as frequencies and percentages. A two-sided *P*-value of less than 0.05 was considered statistically significant. Statistical analyses were performed using SPSS 27.0 (Statistical Package for the Social Sciences)^1^ and R 4.4.0 (the R Foundation).^2^ The variables with more than 20% missing values will be deleted, and missing data will be imputed using the MICE package for multiple imputation.

To avoid overfitting and evaluate the model’s ability to generalize, the sample will be randomly split into a training set (70%) and a test set (30%), with data processing and model development conducted on the training set (18). The optimal tuning parameter (λ) for the Least Absolute Shrinkage and Selection Operator (LASSO) regression analysis will be identified through 10-fold cross-validation, and the most relevant features will be selected using the LASSO method. Subsequently, the selected predictive factors will be incorporated into a multivariate logistic regression analysis, with variables showing a *P*-value < 0.05 included in the final model. The completed model will be visualized through a nomogram.

The clinical value of the prediction model is assessed according to three criteria: discrimination, calibration, and clinical utility. In this study, Area under the curve (AUC) is utilized to evaluate the model’s discriminative power. The calibration curve is employed to measure the alignment between predicted probabilities and actual outcomes. Decision curve analysis (DCA) is applied to assess the model’s clinical utility.

## 3 Results

### 3.1 Participant characteristics

The study ultimately included 803 patients with CRD. The baseline characteristics of the participants are shown in Table 1. Among these participants, 480 were male (59.8%) and 323 were female (40.2%), with 88.2% being married and 60.5% living in rural areas. Among the CRD patients without disability at baseline, after 3 years of follow-up, 196 developed disability, with an incident disability rate of 24.41%. Variables such as age, education level, BMI, and peak expiratory flow rate showed statistical differences (*P* < 0.05). Additionally, we compared the baseline characteristics of the training set (563 cases) and the test set (240 cases), as shown in Supplementary Table 1. With the exception of age, no statistically significant differences were observed in the baseline characteristics between the two groups (*P* > 0.05).

**TABLE 1:** Baseline characteristics of the study population.

Variables	Total (*N* = 803)	Non-disability (*N* = 607)	Disability (*N* = 196)	*P*
Gender (%)				0.004
Female	323 (40.2)	227 (37.4)	96 (49)	
Male	480 (59.8)	380 (62.6)	100 (51)	
Marry (%)				<0.001
Unmarried	95 (11.8)	55 (9.1)	40 (20.4)	
Married	708 (88.2)	552 (90.9)	156 (79.6)	
Residence (%)				0.284
Urban	317 (39.5)	246 (40.5)	71 (36.2)	
Rural	486 (60.5)	361 (59.5)	125 (63.8)	
Self-perceived health status (%)				<0.001
Poor	170 (21.2)	100 (16.5)	70 (35.7)	
Fair	473 (58.9)	368 (60.6)	105 (53.6)	
Good	160 (19.9)	139 (22.9)	21 (10.7)	
Hypertension (%)				0.004
No	558 (69.5)	438 (72.2)	120 (61.2)	
Yes	245 (30.5)	169 (27.8)	76 (38.8)	
Diabetes (%)				0.01
No	736 (91.7)	565 (93.1)	171 (87.2)	
Yes	67 (8.3)	42 (6.9)	25 (12.8)	
Cancer (%)				0.081
No	791 (98.5)	601 (99)	190 (96.9)	
Yes	12 (1.5)	6 (1)	6 (3.1)	
Heart disease (%)				0.005
No	613 (76.3)	478 (78.7)	135 (68.9)	
Yes	190 (23.7)	129 (21.3)	61 (31.1)	
Arthritis (%)				0.002
No	436 (54.3)	348 (57.3)	88 (44.9)	
Yes	367 (45.7)	259 (42.7)	108 (55.1)	
Dyslipidemia (%)				0.857
No	652 (81.2)	492 (81.1)	160 (81.6)	
Yes	151 (18.8)	115 (18.9)	36 (18.4)	
Liver disease (%)				0.392
No	716 (89.2)	538 (88.6)	178 (90.8)	
Yes	87 (10.8)	69 (11.4)	18 (9.2)	
Kidney disease (%)				0.63
No	705 (87.8)	531 (87.5)	174 (88.8)	
Yes	98 (12.2)	76 (12.5)	22 (11.2)	
Digestive disease (%)				0.445
No	514 (64.0)	393 (64.7)	121 (61.7)	
Yes	289 (36.0)	214 (35.3)	75 (38.3)	
Drinking (%)				0.014
No	506 (63.0)	368 (60.6)	138 (70.4)	
Yes	297 (37.0)	239 (39.4)	58 (29.6)	
Smoking (%)				0.353
No	534 (66.5)	409 (67.4)	125 (63.8)	
Yes	269 (33.5)	198 (32.6)	71 (36.2)	
Retire (%)				0.073
No	667 (83.1)	496 (81.7)	171 (87.2)	
Yes	136 (16.9)	111 (18.3)	25 (12.8)	
Education (%)				<0.001
Illiterate	289 (36.0)	198 (32.6)	91 (46.4)	
Primary school	209 (26.0)	152 (25)	57 (29.1)	
Middle school	184 (22.9)	156 (25.7)	28 (14.3)	
High school or beyond	121 (15.1)	101 (16.6)	20 (10.2)	
Memory score	3.5 ± 1.8	3.7 ± 1.8	3.2 ± 1.8	<0.001
Executive score	9.0 (7.0, 10.0)	9.0 (8.0, 10.0)	9.0 (6.0, 10.0)	<0.001
Cognitive score	12.2 ± 3.2	12.5 ± 3.1	11.3 ± 3.4	<0.001
Systolic blood pressure (mmHg)	125.6 ± 19.3	124.4 ± 18.3	129.6 ± 21.7	<0.001
Diastolic blood pressure (mmHg)	73.5 (66.5, 82.0)	73.5 (67.0, 81.5)	74.2 (66.5, 83.5)	0.412
Pulse (/min)	74.6 ± 10.8	74.6 ± 10.7	74.6 ± 11.3	0.99
Hand grip strength (kg)	31.6 ± 10.3	32.6 ± 10.1	28.6 ± 10.2	<0.001
Waist circumference (cm)	85.3 ± 14.2	85.5 ± 14.2	84.7 ± 14.4	0.473
Body mass index (kg/m^2^)	23.3 (20.8, 26.2)	23.4 (21.0, 26.3)	22.6 (20.6, 25.6)	0.046
Peak expiratory flow rate (L/min)	283.4 ± 134.3	297.5 ± 133.5	239.6 ± 127.2	<0.001
CES-D10	6.0 (3.0, 10.0)	6.0 (3.0, 9.0)	8.0 (5.0, 12.2)	<0.001
Life satisfaction	3.4 ± 0.7	3.4 ± 0.7	3.3 ± 0.8	0.286
Sleep time (h)	6.5 ± 1.8	6.5 ± 1.7	6.4 ± 2.0	0.886
Age	61.0 ± 9.0	60.0 ± 8.7	64.1 ± 9.2	<0.001

### 3.2 Predictive model development

**FIGURE 2:**
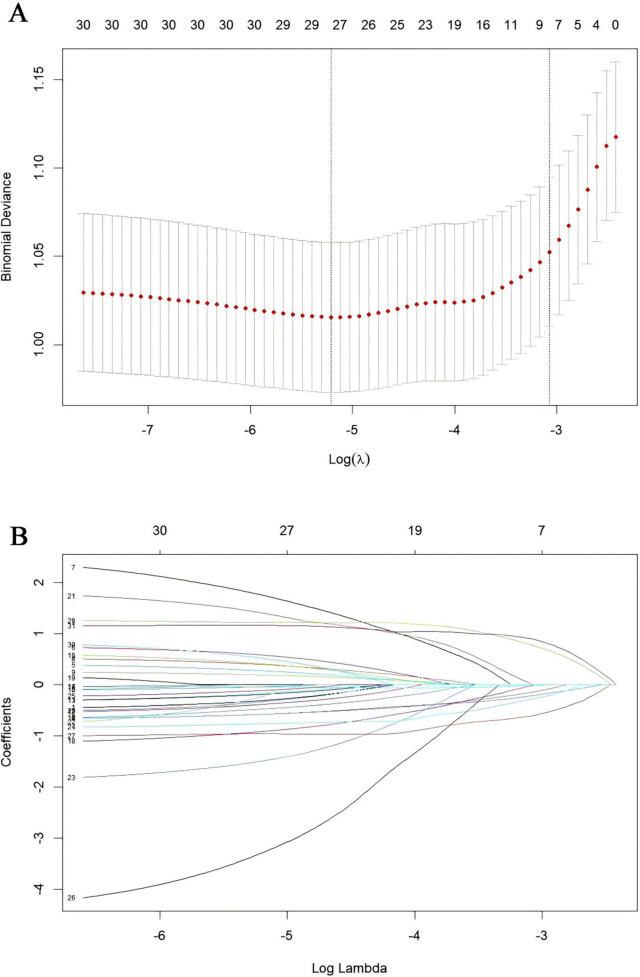
Presentation of the results of the LASSO regression analysis. **(A)** LASSO regression model factor selection. **(B)** LASSO regression model screening variable trajectories.

**FIGURE 3:**
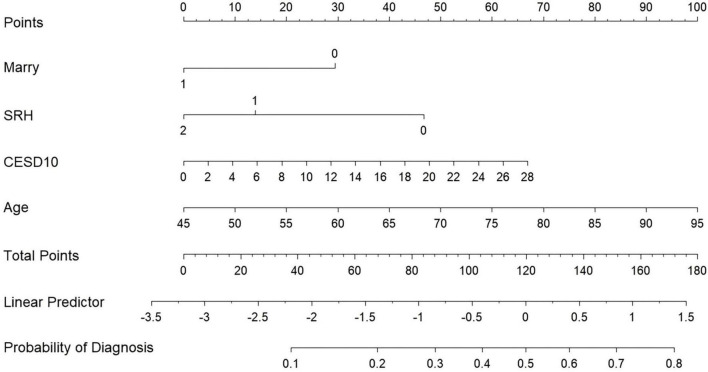
Nomogram.

Our study standardized the training dataset in order to identify the variables most closely associated with disability, employing compressed variable coefficients to mitigate overfitting and address severe multicollinearity. LASSO regression analysis with 10-fold cross-validation was used to determine the optimal penalty parameter λ (Figures 2a,b). Ultimately, eight feature variables were identified: marital status, self-perceived health status, education level, memory, grip strength, peak expiratory flow rate, depressive symptoms, and age. These predictors were then included in a multivariable logistic regression analysis. Of these, marital status (*P* = 0.032), self-perceived health status (*P* < 0.001), depressive symptoms (*P* = 0.005), and age (*P* = 0.008) were incorporated into the nomogram model (Table 2 and Figure 3).

**TABLE 2:** Univariate and multivariate logistic regression analysis.

Variables	Univariate analysis		Multivariate analysis	
	**OR (95%CI)**	***P***	**OR (95%CI)**	***P***
**Marital status**				
Unmarried	1.00 (Reference)		1.00 (Reference)	
Married	0.38 (0.23–0.63)	<0.001	0.53 (0.30–0.95)	0.032
**Self-perceived health status**				
Poor	1.00 (Reference)		1.00 (Reference)	
Fair	0.45 (0.29–0.71)	<0.001	0.43 (0.26–0.72)	0.001
Good	0.25 (0.13–0.48)	<0.001	0.28 (0.13–0.57)	<0.001
**Education**				
Illiterate	1.00 (Reference)		1.00 (Reference)	
Primary school	0.70 (0.43–1.12)	0.132	0.70 (0.42–1.18)	0.181
Middle school	0.34 (0.19–0.61)	<0.001	0.61 (0.32–1.18)	0.143
High school or beyond	0.42 (0.22–0.78)	0.006	0.76 (0.37–1.55)	0.449
Memory score	0.81 (0.73–0.91)	<0.001	0.92 (0.80–1.06)	0.240
Hand grip strength (kg)	0.96 (0.94–0.98)	<0.001	0.99 (0.96–1.01)	0.224
Peak expiratory flow rate (L/min)	0.99 (0.99–0.99)	<0.001	1.00 (1.00–1.00)	0.069
CES-D10	1.08 (1.05–1.12)	<0.001	1.06 (1.02–1.10)	0.005
Age	1.05 (1.03–1.08)	<0.001	1.04 (1.01–1.06)	0.008

### 3.3 Model evaluation

**FIGURE 4:**
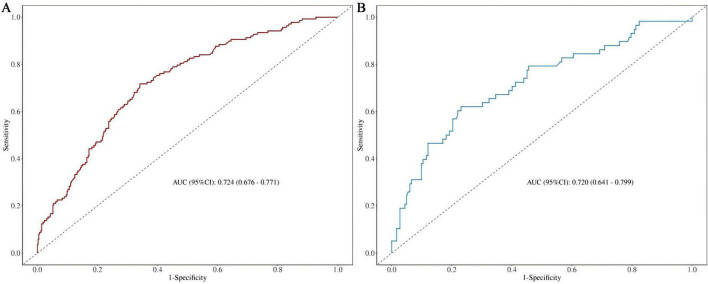
**(A)** Nomogram ROC curves generated from the training dataset. **(B)** Nomogram ROC curves generated using the test dataset.

The discriminatory ability of the nomogram model was evaluated using the AUC value. As depicted in Figure 4 and Supplementary Table 2, the AUC for the training set (Figure 4A) was 0.724 (95% CI: 0.676-0.771), with an accuracy of 0.671, sensitivity of 0.659, and specificity of 0.710. In the test set (Figure 4B), the AUC was 0.720 (95% CI: 0.641-0.799), with an accuracy of 0.700, sensitivity of 0.725, and specificity of 0.621. These results demonstrate that the model possesses robust discriminative ability and predictive value, effectively distinguishing between disabled and non-disabled patients. Furthermore, we constructed a Random Forest model to compare predictive performance. The results indicated a significant discrepancy in the AUC values between the training and testing sets on the ROC curve (training set: 1, testing set: 0.75), highlighting a clear overfitting issue with the Random Forest model (Supplementary Figure 1). As a result, we opted for the more stable logistic regression model as the final model.

**FIGURE 5:**
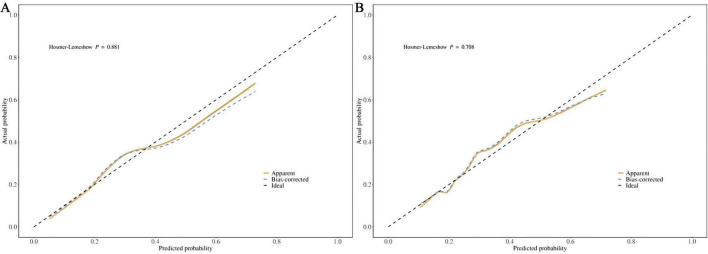
**(A)** Calibration curve for the training dataset. **(B)** Calibration curve for the test dataset.

The calibration curve and the Hosmer-Lemeshow goodness-of-fit test were employed to evaluate the alignment between the observed outcomes and the predicted probabilities. The calibration plot based on the multivariable logistic regression model, shown in Figures 5a,b, demonstrates good agreement between the observed and predicted values for both the training and test sets, indicating a good level of consistency. The *p*-values of the Hosmer-Lemeshow goodness-of-fit test exceeded 0.05, indicating that the nomogram model demonstrates a good fit for both the training set (χ^2^ = 3.7204, df = 8, *p* = 0.8814) and the test set (χ^2^ = 5.4584, df = 8, *p* = 0.7076).

**FIGURE 6:**
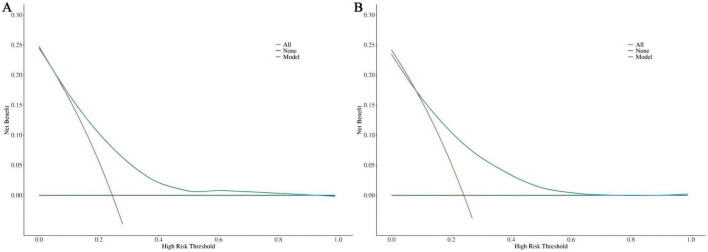
**(A)** DCA curve for the training dataset. **(B)** DCA curve for the test dataset.

DCA was utilized to assess the clinical validity of the model. According to the DCA for the training (Figure 6A) and test (Figure 6B) sets, interventions guided by the predictive model demonstrated excellent performance, except for a small range of low-preference thresholds. The results revealed that the nomogram model yielded substantial net benefits in both the training and test sets, indicating that our model possesses strong predictive accuracy and clinical efficacy in forecasting the risk of incident disability.

## 4 Discussion

CRD and disability are prevalent conditions that profoundly impact patients’ quality of life. Previous studies have indicated that the incidence of disability is considerably higher among CRD patients, ranging from 7.4 to 49.8% (19), a finding that aligns with the 24.41% disability incidence observed in this study. Disability restricts individuals’ access to quality education, employment, and income, placing them at heightened risk of poverty, lower socioeconomic status, inadequate housing, and limited access to nutritious food and healthcare services (20, 21). These factors contribute to the deterioration of health and an increased reliance on others. Thus, identifying high-risk individuals within the CRD population is essential for preventing disability and its associated adverse outcomes.

In our study, we developed a nomogram model using data from CHARLS to aid clinicians in evaluating the risk of disability in CRD patients. The nomogram calculates scores for each feature using the scale at the top, allowing the risk of disability in CRD patients to be estimated by drawing a vertical line from the total points axis to the risk axis. Our final model incorporates four predictors: marital status, self-perceived health status, depressive symptoms, and age. Among these, being unmarried, reporting poor health, having a higher CES-D10 score, and older age are identified as risk factors for disability in CRD patients. These factors have been well-documented in previous studies.

Research has demonstrated that unmarried individuals are more susceptible to disability than their married counterparts (22). Marriage confers a protective effect against functional impairment, as individuals with disabilities often impose emotional strain on those around them, particularly their spouses. This dynamic can hinder the ability of those with disabilities to maintain close relationships or stable marriages. Furthermore, unmarried individuals lack the caregiving and support typically provided by a spouse or family, which may render them more vulnerable to feelings of loneliness and helplessness, thereby increasing their risk of health issues or disability. In contrast, married individuals are more likely to benefit from familial or social support in managing chronic conditions, such as respiratory diseases, which tends to result in a lower incidence of illness. Previous sociological studies have shown that married individuals generally experience better health outcomes and lower mortality rates (23, 24). Hence, it is crucial to implement supportive measures for unmarried individuals, including the establishment of social support systems, mental health interventions, enhanced health management education, economic assistance, and the development of favorable social policies. Such initiatives can mitigate health risks, improve quality of life, and reduce the incidence of disability among unmarried individuals.

Self-perceived health status refers to an individual’s personal evaluation of their health, grounded in their own feelings and experiences (25). Those who self-report poorer health are more likely to face long-term health challenges, the progression of chronic diseases, and physical disability. Research has demonstrated that both global and severe disability are strongly linked to various morbidities and self-assessments of diminished health (26). Mental health conditions, such as depression and anxiety, play a significant role in shaping how individuals perceive their health. Depressed individuals often view their health as worse, and these negative emotions may foster the onset of disability or other health problems through mechanisms such as heightened inflammation and immune system dysfunction (27). Moreover, individuals with poorer self-rated health are typically burdened with a higher prevalence of chronic diseases, such as hypertension, diabetes, and cardiovascular conditions, which frequently lead to impaired physical function and disability. Rijken et al. highlighted that the combination of diabetes, cardiovascular diseases, and chronic respiratory conditions increases the risk of physical disability (28).

We employed the CES-D10 scale to evaluate depressive symptoms and discovered that depression serves as a significant predictor of disability in patients with CRD. This result aligns with previous studies, which have demonstrated that the coexistence of chronic respiratory diseases and depression is linked to higher levels of disability (29). Additional research also suggests that both depression and somatic symptoms are independently associated with disability (30). Individuals with mental health conditions are more prone to experiencing functional limitations than those without such conditions. From a physiological standpoint, depressed patients often display elevated levels of inflammation, which is closely tied to various conditions, including cardiovascular diseases, diabetes, and arthritis. Chronic inflammation, in conjunction with CRD, contributes to the decline in physical function, leading to or aggravating disability (27). Moreover, physical symptoms related to depression in CRD patients, such as dyspnea and fatigue, can further hinder physical function, limiting their capacity to perform daily activities (31). Psychologically, individuals with depression often experience profound fatigue, a loss of interest, and may struggle to carry out basic daily tasks such as eating, dressing, and bathing (32). Furthermore, they may reduce their social and physical activities due to a persistent low mood, and prolonged inactivity can lead to muscle atrophy, joint stiffness, and other complications, further impairing their ability to care for themselves (33). Healthcare providers should remain attentive to the mental health of these patients, stay vigilant for signs of negative emotions, and implement preventive strategies to avert the onset of depression and disability.

Age is closely linked to the risk of disability in patients with CRD. As individuals age, their physiological functions gradually decline, often resulting in muscle atrophy and decreased bone density—key physiological mechanisms contributing to disability (34). The degeneration of muscles and bones renders middle-aged and elderly individuals more vulnerable to injuries such as falls and fractures, which can lead to long-term functional impairment. Older adults, particularly those with chronic respiratory diseases, are also more susceptible to cognitive impairments, including memory loss, language difficulties, and a decline in daily living skills, often necessitating care and assistance from others (35). Furthermore, with advancing age, the prevalence of comorbid chronic conditions, such as coronary artery disease and stroke, rises significantly (36). The onset of these diseases can profoundly affect a patient’s daily life, leading to disability.

Prior to this, we found no reports of predictive models for new-onset disability in patients with CRD. In this study, we identified marital status, self-perceived health, depressive symptoms, and age as the major factors predicting disability in CRD patients. The model constructed based on these four variables showed an AUC of 0.724 (95% CI: 0.676-0.771) in the training set and 0.720 (95% CI: 0.641-0.799) in the test set, with good accuracy, sensitivity, and specificity, indicating strong discriminative ability of the model. Although an AUC around 0.7 may not be considered exceptionally strong in terms of discriminative ability, in the medical field, particularly in chronic disease prediction, given its significance for early screening, such an AUC may hold greater practical value (37, 38). At the same time, we incorporated the Random Forest machine learning algorithm for comparison, which revealed a clear overfitting effect. Overfitted predictive models typically perform poorly in real clinical settings. This issue is particularly prevalent in studies with smaller sample sizes, especially those based on machine learning and deep learning algorithms. Therefore, for the purpose of this study, the logistic regression-based model remains the optimal solution for clinical translation. The DCA demonstrated that the model provided substantial net benefit across most threshold probability ranges, suggesting its good clinical applicability. Furthermore, calibration of the model was assessed through the use of calibration curves, and both the training and test sets showed a Hosmer-Lemeshow goodness-of-fit *p*-value greater than 0.05, indicating good calibration and predictive accuracy. As a highly efficient and accurate assessment tool, our predictive model can aid physicians in identifying high-risk CRD populations, offering both theoretical foundation and empirical evidence for early prevention and intervention strategies. The model exhibits outstanding clinical applicability and can effectively identify patients at heightened risk of disability.

The current study has several limitations. Firstly, certain potential predictive factors, such as blood gas analysis, pulmonary function test results, and the history of acute exacerbations of respiratory diseases, were not available in the CHARLS database. Secondly, our model was developed and validated using data from the Chinese population, and further validation using larger sample sizes and external cohort data is necessary to assess whether the findings can be generalized to other regions and countries. Thirdly, the diagnosis of CRD was reliant on self-reported data, which may have resulted in an underestimation of its true prevalence. These selection and information biases may have affected the representativeness of the study sample. And the patient’s health status, social support, and environmental factors may change over time, and these fluctuations could significantly impact the risk of disability. However, our model struggles to dynamically capture these changes.

## 5 Conclusion

Our study designed and validated a nomogram model to predict the 3-year risk of new-onset disability in patients with CRD. Our model, which incorporates marital status, self-perceived health, depressive symptoms, and age, demonstrated excellent clinical applicability, and efficiency. This model holds significant value for the early identification and intervention of CRD patients at potential risk for disability.

## Data Availability

Publicly available datasets were analyzed in this study. This data can be found at: CHARLS database website (https://charls.pku.edu.cn).

## Footnotes

1
https://www.ibm.com/products/spss-statistics

2
http://www.R-project.org

## References

[1] Han Y Wang S. Disability risk prediction model based on machine learning among Chinese healthy older adults: Results from the China Health and Retirement Longitudinal Study. *Front Public Health.* (2023) 11:1271595. 10.3389/fpubh.2023.1271595 38026309 PMC10665855

[2] GBD 2019 Chronic Respiratory Diseases Collaborators. Global burden of chronic respiratory diseases and risk factors, 1990-2019: An update from the Global Burden of Disease Study 2019. *EClinicalMedicine.* (2023) 59:101936. 10.1016/j.eclinm.2023.101936 37229504 PMC7614570

[3] Ogunbayo O Russell S Newham J Heslop-Marshall K Netts P Hanratty B Understanding the factors affecting self-management of COPD from the perspectives of healthcare practitioners: A qualitative study. *NPJ Prim Care Respir Med.* (2019) 27:54. 10.1038/s41533-017-0054-6 28924245 PMC5603550

[4] Bahra N Amara B Bourkhime H El Yaagoubi S Othmani N Tachfouti N Quality of life and its determinants in patients with chronic respiratory diseases in the Fes-Meknes region, Morocco. *Monaldi Arch Chest Dis.* (2024) [Online ahead of print]. 10.4081/monaldi.2024.2964 39283027

[5] Lawton M Brody E. Assessment of older people: Self-maintaining and instrumental activities of daily living. *Gerontologist.* (1969) 9:179–86. 10.1093/geront/9.3_Part_1.179 5349366

[6] Katz S Ford A Moskowitz R Jackson B Jaffe M. Studies of illness in the aged. the index of adl: A standardized measure of biological and psychosocial function. *JAMA.* (1963) 185:914–9. 10.1001/jama.1963.03060120024016 14044222

[7] Koyano W Shibata H Haga H Suyama Y. Prevalence and outcome of low ADL and incontinence among the elderly: Five years follow-up in a Japanese urban community. *Arch Gerontol Geriatr.* (1986) 5:197–206. 10.1016/0167-4943(86)90022-1 2948464

[8] Ćwirlej-Sozańska A Wiśniowska-Szurlej A Wilmowska-Pietruszyńska A Sozański B. Determinants of ADL and IADL disability in older adults in Southeastern Poland. *BMC Geriatr.* (2019) 19:297. 10.1186/s12877-019-1319-4 31672121 PMC6824102

[9] Sjölund B Wimo A Engström M von Strauss E. Incidence of ADL disability in older persons, physical activities as a protective factor and the need for informal and formal care–results from the SNAC-N Project. *PLoS One.* (2015) 10:e0138901. 10.1371/journal.pone.0138901 26407207 PMC4583409

[10] Stuck A Walthert J Nikolaus T Büla C Hohmann C Beck J. Risk factors for functional status decline in community-living elderly people: A systematic literature review. *Soc Sci Med.* (1999) 48:445–69. 10.1016/s0277-9536(98)00370-0 10075171

[11] Niu Y Li N Jin C Chen D Yang Y Ding H. Activity outside the home, environmental barriers, and healthy aging for community-dwelling elderly individuals in China. *Biosci Trends.* (2017) 11:603–5. 10.5582/bst.2017.01266 29151555

[12] Wang D Yao J Zirek Y Reijnierse E Maier A. Muscle mass, strength, and physical performance predicting activities of daily living: A meta-analysis. *J Cachexia Sarcopenia Muscle.* (2020) 11:3–25. 10.1002/jcsm.12502 31788969 PMC7015244

[13] Luo X Pietrobon R Sun S Liu G Hey L. Estimates and patterns of direct health care expenditures among individuals with back pain in the United States. *Spine.* (2004) 29:79–86. 10.1097/01.BRS.0000105527.13866.0F 14699281

[14] Zhao Y Hu Y Smith J Strauss J Yang G. Cohort profile: The China Health and Retirement Longitudinal Study (CHARLS). *Int J Epidemiol.* (2014) 43:61–8. 10.1093/ije/dys203 23243115 PMC3937970

[15] Li M Yang Y Pang L Wu M Wang Z Fu Y Gender-specific associations between activities of daily living disability and depressive symptoms among older adults in China: Evidence from the China Health and Retirement Longitudinal Study. *Arch Psychiatr Nurs.* (2019) 33:160–6. 10.1016/j.apnu.2019.08.010 31753223

[16] Carmona-Torres J Rodríguez-Borrego M Laredo-Aguilera J López-Soto P Santacruz-Salas E Cobo-Cuenca A. Disability for basic and instrumental activities of daily living in older individuals. *PLoS One.* (2019) 14:e0220157. 10.1371/journal.pone.0220157 31348797 PMC6660130

[17] Yan Y Du Y Li X Ping W Chang Y. Physical function, ADL, and depressive symptoms in Chinese elderly: Evidence from the CHARLS. *Front Public Health.* (2023) 11:1017689. 10.3389/fpubh.2023.1017689 36923048 PMC10010774

[18] Xu Y Goodacre R. On splitting training and validation set: A comparative study of cross-validation, bootstrap and systematic sampling for estimating the generalization performance of supervised learning. *J Anal Test.* (2018) 2:249–62. 10.1007/s41664-018-0068-2 30842888 PMC6373628

[19] Lisy K Campbell J Tufanaru C Moola S Lockwood C. The prevalence of disability among people with cancer, cardiovascular disease, chronic respiratory disease and/or diabetes: A systematic review. *Int J Evid Based Healthc.* (2018) 16:154–66. 10.1097/XEB.0000000000000138 29608458

[20] Adams M Augustyns N Janssens H Vriesacker B Van Hal G. What socio-demographic factors influence poverty and financial health care access among disabled people in Flanders: A cross-sectional study. *Arch Public Health.* (2014) 72:5. 10.1186/2049-3258-72-5 24521283 PMC3930006

[21] Bechange S Jolley E Jeyam A Okello G Wekesa B Schmidt E. Disability and labour market participation among smallholder farmers in Western Kenya. *PLoS One.* (2024) 19:e0306458. 10.1371/journal.pone.0306458 38968175 PMC11226002

[22] Zhang T Huang Y Liu Z Chen H. Distribution and risk factors of disability attributed to personality disorders: A national cross-sectional survey in China. *Chin Med J.* (2016) 129:1765–71. 10.4103/0366-6999.186649 27453222 PMC4976561

[23] Wong C Kwok C Narain A Gulati M Mihalidou A Wu P Marital status and risk of cardiovascular diseases: A systematic review and meta-analysis. *Heart.* (2018) 104:1937–48. 10.1136/heartjnl-2018-313005 29921571

[24] Bulloch A Williams J Lavorato D Patten S. The depression and marital status relationship is modified by both age and gender. *J Affect Disord.* (2017) 223:65–8. 10.1016/j.jad.2017.06.007 28732242

[25] Park Y Kim S. Factors associated with clinical nurses’ preconception health behavior in Korea: A cross-sectional survey. *Womens Health Nurs.* (2024) 30:79–89. 10.4069/whn.2024.03.08 38650329 PMC11073552

[26] Montoro Pazzini Watfe G Fajersztajn L Ribeiro E Rossi Menezes P Scazufca M. Prevalence of older adult disability and primary health care responsiveness in low-income communities. *Life.* (2020) 10:133. 10.3390/life10080133 32764217 PMC7460338

[27] Morin R Nelson C Bickford D Insel P Mackin R. Somatic and anxiety symptoms of depression are associated with disability in late life depression. *Aging Ment Health.* (2020) 24:1225–8. 10.1080/13607863.2019.1597013 30945553 PMC9183787

[28] Rijken M van Kerkhof M Dekker J Schellevis F. Comorbidity of chronic diseases: Effects of disease pairs on physical and mental functioning. *Qual Life Res.* (2005) 14:45–55. 10.1007/s11136-004-0616-2 15789940

[29] Yokota R Van der Heyden J Nusselder W Robine J Tafforeau J Deboosere P Impact of chronic conditions and multimorbidity on the disability burden in the older population in belgium. *J Gerontol A Biol Sci Med Sci.* (2016) 71:903–9. 10.1093/gerona/glv234 26774118

[30] Gontijo Guerra S Berbiche D Vasiliadis H. Changes in instrumental activities of daily living functioning associated with concurrent common mental disorders and physical multimorbidity in older adults. *Disabil Rehabil.* (2021) 43:3663–71. 10.1080/09638288.2020.1745303 32255362

[31] Verhaak P Dekker J de Waal M van Marwijk H Comijs H. Depression, disability and somatic diseases among elderly. *J Affect Disord.* (2014) 167:187–91. 10.1016/j.jad.2014.05.057 24992026

[32] Kong D Solomon P Dong X. Depressive symptoms and onset of functional disability over 2 years: A prospective cohort study. *J Am Geriatr Soc.* (2019) 67:S538–44. 10.1111/jgs.15801 31403199 PMC9942515

[33] Noguchi T Saito M Aida J Cable N Tsuji T Koyama S Association between social isolation and depression onset among older adults: A cross-national longitudinal study in England and Japan. *BMJ Open.* (2021) 11:e045834. 10.1136/bmjopen-2020-045834 33737442 PMC7978252

[34] Li A Sun Y Li M Wang D Ma X. Effects of elastic band resistance training on the physical and mental health of elderly individuals: A mixed methods systematic review. *PLoS One.* (2024) 19:e0303372. 10.1371/journal.pone.0303372 38739588 PMC11090353

[35] Li J Lin S Yan X Wei Y Yang F Pei L. Cross-country comparison of income-related inequality in physical functional disability among middle-aged and older adults: Evidence from 33 countries. *J Glob Health.* (2023) 13:04053. 10.7189/jogh.13.04053 37204132 PMC10197408

[36] Wang Z Peng W Li M Li X Yang T Li C Association between multimorbidity patterns and disability among older people covered by long-term care insurance in Shanghai, China. *BMC Public Health.* (2021) 21:418. 10.1186/s12889-021-10463-y 33639902 PMC7912511

[37] Li E Ai F Liang C. A machine learning model to predict the risk of depression in US adults with obstructive sleep apnea hypopnea syndrome: A cross-sectional study. *Front Public Health.* (2023) 11:1348803. 10.3389/fpubh.2023.1348803 38259742 PMC10800603

[38] Sulaieva O Yerokhovych V Zemskov S Komisarenko I Gurianov V Pankiv V The impact of war on people with type 2 diabetes in Ukraine: A survey study. *EClinicalMedicine.* (2024) 79:103008. 10.1016/j.eclinm.2024.103008 39791105 PMC11714670

