# Supplementary figures and images for: New-onset disability risk prediction model for chronic respiratory disease patients: the first longitudinal evidence from CHARLS
Source: Front Med (Lausanne). 2025 May 20;12:1545387. doi: 10.3389/fmed.2025.1545387 (PMC12129796; doi:10.3389/fmed.2025.1545387)

**Supplementary Figure S1** Random Forest Algorithm: ROC Curves for the Training and Testing Sets.

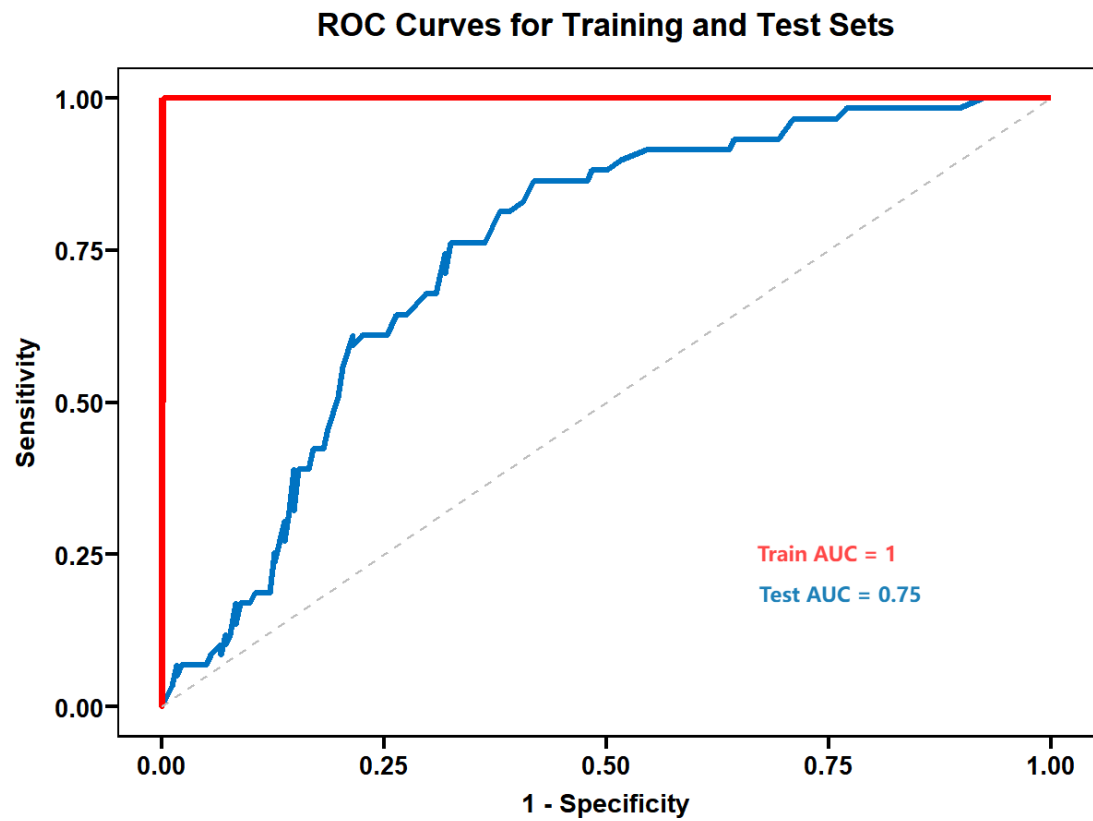

Supplement: Supplementary file 1 [file Image_1.pdf]
