# Supplementary material for: New-onset disability risk prediction model for chronic respiratory disease patients: the first longitudinal evidence from CHARLS
Source: Front Med (Lausanne). 2025 May 20;12:1545387. doi: 10.3389/fmed.2025.1545387 (PMC12129796; doi:10.3389/fmed.2025.1545387)
Supplement: Supplementary file 2 [file Supplementary_file_1.docx]

**Supplementary Table S1** Training set and Test set variability analysis.

| **Variables** | **Total (N = 803)** | **Training (N = 563)** | **Test (N = 240)** | ***P*** |
| --- | --- | --- | --- | --- |
| Disability (%) |  |  |  | 0.917 |
| No | 607 (75.6) | 425 (75.5) | 182 (75.8) |  |
| Yes | 196 (24.4) | 138 (24.5) | 58 (24.2) |  |
| Gender (%) |  |  |  | 0.31 |
| Female | 323 (40.2) | 220 (39.1) | 103 (42.9) |  |
| Male | 480 (59.8) | 343 (60.9) | 137 (57.1) |  |
| Marry (%) |  |  |  | 0.127 |
| Unmarried | 95 (11.8) | 73 (13) | 22 (9.2) |  |
| Married | 708 (88.2) | 490 (87) | 218 (90.8) |  |
| Residence (%) |  |  |  | 0.783 |
| Urban | 317 (39.5) | 224 (39.8) | 93 (38.8) |  |
| Rural | 486 (60.5) | 339 (60.2) | 147 (61.3) |  |
| Self-perceived health status (%) |  |  |  | 0.939 |
| Poor | 170 (21.2) | 119 (21.1) | 51 (21.2) |  |
| Fair | 473 (58.9) | 330 (58.6) | 143 (59.6) |  |
| Good | 160 (19.9) | 114 (20.2) | 46 (19.2) |  |
| Hypertension (%) |  |  |  | 0.527 |
| No | 558 (69.5) | 395 (70.2) | 163 (67.9) |  |
| Yes | 245 (30.5) | 168 (29.8) | 77 (32.1) |  |
| Diabetes (%) |  |  |  | 0.994 |
| No | 736 (91.7) | 516 (91.7) | 220 (91.7) |  |
| Yes | 67 ( 8.3) | 47 (8.3) | 20 (8.3) |  |
| Cancer (%) |  |  |  | 0.199 |
| No | 791 (98.5) | 557 (98.9) | 234 (97.5) |  |
| Yes | 12 ( 1.5) | 6 (1.1) | 6 (2.5) |  |
| Heart disease (%) |  |  |  | 0.26 |
| No | 613 (76.3) | 436 (77.4) | 177 (73.8) |  |
| Yes | 190 (23.7) | 127 (22.6) | 63 (26.2) |  |
| Arthritis (%) |  |  |  | 0.839 |
| No | 436 (54.3) | 307 (54.5) | 129 (53.8) |  |
| Yes | 367 (45.7) | 256 (45.5) | 111 (46.2) |  |
| Dyslipidemia (%) |  |  |  | 0.674 |
| No | 652 (81.2) | 455 (80.8) | 197 (82.1) |  |
| Yes | 151 (18.8) | 108 (19.2) | 43 (17.9) |  |
| Liver disease (%) |  |  |  | 0.804 |
| No | 716 (89.2) | 501 (89) | 215 (89.6) |  |
| Yes | 87 (10.8) | 62 (11) | 25 (10.4) |  |
| Kidney disease (%) |  |  |  | 0.267 |
| No | 705 (87.8) | 499 (88.6) | 206 (85.8) |  |
| Yes | 98 (12.2) | 64 (11.4) | 34 (14.2) |  |
| Digestive disease (%) |  |  |  | 0.062 |
| No | 514 (64.0) | 372 (66.1) | 142 (59.2) |  |
| Yes | 289 (36.0) | 191 (33.9) | 98 (40.8) |  |
| Drinking (%) |  |  |  | 0.606 |
| No | 506 (63.0) | 358 (63.6) | 148 (61.7) |  |
| Yes | 297 (37.0) | 205 (36.4) | 92 (38.3) |  |
| Smoking (%) |  |  |  | 0.671 |
| No | 534 (66.5) | 377 (67) | 157 (65.4) |  |
| Yes | 269 (33.5) | 186 (33) | 83 (34.6) |  |
| Retire (%) |  |  |  | 0.192 |
| No | 667 (83.1) | 474 (84.2) | 193 (80.4) |  |
| Yes | 136 (16.9) | 89 (15.8) | 47 (19.6) |  |
| Education (%) |  |  |  | 0.732 |
| Illiterate | 289 (36.0) | 206 (36.6) | 83 (34.6) |  |
| Primary school | 209 (26.0) | 145 (25.8) | 64 (26.7) |  |
| Middle school | 184 (22.9) | 124 (22) | 60 (25) |  |
| High school or beyond | 121 (15.1) | 88 (15.6) | 33 (13.8) |  |
| Memeory score | 3.5 ± 1.8 | 3.5 ± 1.8 | 3.6 ± 1.9 | 0.325 |
| Executive score | 9.0 (7.0, 10.0) | 9.0 (8.0, 10.0) | 9.0 (7.0, 10.0) | 0.239 |
| Cognitive score | 12.2 ± 3.2 | 12.2 ± 3.1 | 12.2 ± 3.4 | 0.873 |
| Systolic blood pressure (mmHg) | 125.6 ± 19.3 | 126.0 ± 19.5 | 124.8 ± 18.9 | 0.444 |
| Diastolic blood pressure (mmHg) | 73.5 (66.5, 82.0) | 74.0 (67.0, 82.2) | 73.2 (66.0, 81.5) | 0.514 |
| Pulse (/min) | 74.6 ± 10.8 | 74.7 ± 11.1 | 74.3 ± 10.3 | 0.638 |
| Hand grip strength (kg) | 31.6 ± 10.3 | 31.3 ± 10.2 | 32.4 ± 10.5 | 0.167 |
| Waist circumference (cm) | 85.3 ± 14.2 | 85.5 ± 13.8 | 85.0 ± 15.3 | 0.669 |
| Body mass index (kg/m^2^) | 23.3 (20.8, 26.2) | 23.3 (20.8, 26.1) | 23.2 (20.7, 26.3) | 0.856 |
| Peak expiratory flow rate（L/min） | 283.4 ± 134.3 | 278.7 ± 134.0 | 294.2 ± 134.4 | 0.135 |
| CES-D10 | 6.0 (3.0, 10.0) | 6.0 (3.0, 10.0) | 7.0 (3.0, 10.0) | 0.278 |
| Life satisfaction | 3.4 ± 0.7 | 3.4 ± 0.7 | 3.4 ± 0.7 | 0.987 |
| Sleep time (h) | 6.5 ± 1.8 | 6.5 ± 1.9 | 6.4 ± 1.7 | 0.86 |
| Age | 61.0 ± 9.0 | 61.5 ± 9.1 | 59.9 ± 8.6 | 0.023 |
