# Supplementary material for: New-onset disability risk prediction model for chronic respiratory disease patients: the first longitudinal evidence from CHARLS
Source: Front Med (Lausanne). 2025 May 20;12:1545387. doi: 10.3389/fmed.2025.1545387 (PMC12129796; doi:10.3389/fmed.2025.1545387)
Supplement: Supplementary file 3 [file Supplementary_file_2.docx]

**Supplementary Table S2** Detailed performance metrics of model for training and test sets

| **Data** | **AUC (95%CI)** | **Accuracy (95%CI)** | **Sensitivity (95%CI)** | **Specificity (95%CI)** | **PPV (95%CI)** | **NPV (95%CI)** | **cut off** |
| --- | --- | --- | --- | --- | --- | --- | --- |
|  |  |  |  |  |  |  |  |
| Train | 0.724 (0.676-0.771) | 0.671 (0.631-0.710) | 0.659 (0.614 - 0.704) | 0.710 (0.634 - 0.786) | 0.875 (0.839 - 0.911) | 0.403 (0.342 - 0.465) | 0.237 |
| Test | 0.720 (0.641-0.799) | 0.700 (0.638-0.757) | 0.725 (0.660 - 0.790) | 0.621 (0.496 - 0.746) | 0.857 (0.802 - 0.912) | 0.419 (0.314 - 0.523) | 0.237 |

AUC: Area under the curve; PPV: Positive predictive value; NPV: Negative predictive value
